# The Benefits of Family Screening in Rare Diseases: Genetic Testing Reveals 165 New Cases of Fabry Disease among At-Risk Family Members of 83 Index Patients

**DOI:** 10.3390/genes13091619

**Published:** 2022-09-09

**Authors:** Sergey Moiseev, Ekaterina Tao, Alexey Moiseev, Nikolay Bulanov, Ekaterina Filatova, Victor Fomin, Dominique P. Germain

**Affiliations:** 1 Tareev Clinic of Internal Disease, Sechenov First Moscow State Medical University, 119991 Moscow, Russia; 2 Faculty of Medicine, Lomonosov Moscow State University, 119991 Moscow, Russia; 3 Geneo Referral Centre for Fabry Disease, Filière G2M, MetabERN European Reference Network, Paris-Saclay University, 92380 Garches, France; 4 Second Department of Internal Medicine, First Faculty of Medicine, Charles University, 12808 Prague, Czech Republic; 5 Faculty of Medicine, University of Puthisastra, Phnom Penh 12211, Cambodia; 6 Division of Medical Genetics, University of Versailles, 78180 Montigny, France

**Keywords:** rare diseases, Fabry disease, family screening, cascade genotyping, early diagnosis

## Abstract

Background: Fabry disease (FD, OMIM #301500) is a rare, progressive, X-linked, inherited genetic disease caused by a functional deficiency of lysosomal α-galactosidase, leading to the accumulation of glycosphingolipids in virtually all of the body’s cell types and fluids. Patients with rare genetic diseases and non-specific symptoms often experience substantial diagnostic delays, which can negatively impact the prompt initiation of treatment. If FD is not treated specifically, end organ damage (such as chronic renal failure, hypertrophic cardiomyopathy with arrhythmia, and strokes) impairs quality of life and reduces life expectancy. Patients and Methods: For 83 consecutive patients with FD referred to the Russian reference center for lysosomal storage diseases, family trees were built and genetic testing (cascade genotyping) was offered to family members. Results: The pathogenic *GLA* variant associated with FD was identified for all 83 probands. Family testing using cascade genotyping enabled the identification of 165 additional cases of FD among the tested 331 at-risk family members. Discussion: This is the first study to have described family screening in a large Russian cohort of patients with FD and chronic kidney disease. Raising awareness of FD among clinicians is important for earlier diagnosis and specific treatment.

## 1. Introduction

Fabry disease (OMIM #301500) is a rare, X-linked, lysosomal disorder caused by pathogenic variants in the *GLA* gene (HUGO Gene Nomenclature Committee ID: 4296; Gene Entrez: 2717; NCBI reference sequence: NM_000169.3) [1,2]. The genetic defect results in the absence of (or a substantial decrease in) α-galactosidase activity (AGAL, Enzyme Commission number: EC 3.2.1.22; UniProt ID: P06280) [3,4,5] and thus the accumulation of glycosphingolipids (mainly globotriosylceramide, also referred to as GL3 and Gb_3_) and its deacylated derivative (globotriaosylsphingosine, also referred to as lyso-Gb_3_) [6] in most of the body’s cell types and fluids. This accumulation progressively and irreversibly damages vital organs, including the kidney, the heart, and the brain.

Early signs and symptoms of Fabry disease include neuropathic pain [7], angiokeratoma, hypohidrosis, and gastrointestinal symptoms (nausea, vomiting, abdominal pain, and episodes of diarrhea) from childhood or adolescence onwards, which precede overt renal impairment [5,8], left ventricular hypertrophy [9], and/or recurrent strokes or transient ischemic attacks [10,11]. The clinical expression of Fabry disease is usually more severe in hemizygous males than in heterozygous females. However, females also can exhibit both early and late signs and symptoms of Fabry disease as a result of skewed X-chromosome inactivation, which can favor the expression of the mutant allele [12].

Given the effectiveness of long-term enzyme replacement therapy [13,14,15] and treatment with small-molecule pharmacological chaperones [16], timely diagnosis is the key to the successful treatment of Fabry disease. Disease-specific therapies can reduce levels of neuropathic pain and prevent or slow down organ damage [17]. However, FD is often diagnosed late—sometimes decades late—because clinicians are not sufficiently aware of rare diseases, or because patients with a later-onset Fabry disease phenotype (often affecting a single organ system, commonly the heart) do not display alerting signs and symptoms. Moreover, the initial symptoms of classic Fabry disease can also be non-specific (e.g., gastrointestinal disorders and autonomic neuropathy), misleading (e.g., recurrent, unexplained fever), or overlooked (a skin rash and a keratopathy, cornea verticillata).

Undiagnosed patients with Fabry disease can be detected by screening at-risk populations, such as patients with end-stage renal disease undergoing dialysis or kidney transplantation [18,19,20,21,22,23,24,25,26], patients with unexplained left ventricular hypertrophy [27,28,29,30], and young adults with a history of stroke or transient ischemic attack [31]. The diagnostic yield of these expensive screening programs is low: from 0–0.2% in dialysis units to 0.5–0.9% in patients with unexplained left ventricular hypertrophy [32]. Moreover, diagnosed patients in at-risk groups often have late-stage renal disease or cerebrovascular disease with irreversible organ damage, which has a negative impact on clinical outcomes. In certain screening settings (e.g., adult patients with early, undifferentiated arthritis or children with chronic pain in the distal limbs), the diagnostic yield is essentially zero. Nevertheless, screening protocols for Fabry disease in at-risk patients also pave the way to family screening; affected relatives (notably children and young adults) can then benefit from earlier treatment and genetic counselling [33]. Testing the family of newly diagnosed patients with Fabry disease can also improve a screening protocol’s cost effectiveness [33].

The objectives of the present study were to evaluate the feasibility and effectiveness of family genetic testing for index patients with Fabry disease and to identify obstacles to this testing.

## 2. Patients and Methods

We performed a retrospective study of 83 consecutive patients with Fabry disease, consulting at a Russian reference center for lysosomal diseases (Tareev Clinic of Internal Diseases, Moscow, Russia). All participants gave their written, informed consent to use of their personal medical data. The study was conducted in accordance with the tenets of the Declaration of Helsinki and was approved by the local institutional review board (Sechenov University, Moscow, Russia) on 24 January 2021.

All patients were asked about other family members who were potentially affected by Fabry disease, in view of the X-linked pattern of inheritance [34]. If necessary, patient’s relatives were asked to provide more detailed information about the family. For each proband, a family tree was constructed and analyzed with regard to: the total number of family members at risk of inheriting the pathogenic variant, the number of relatives screened for Fabry disease, and the number of relatives diagnosed with Fabry disease. Deceased family members were included in the study only if Fabry disease had been unambiguously diagnosed prior to death.

The diagnostic criteria for Fabry disease included low or absent AGAL activity and a pathogenic variant in the *GLA* gene combined with at least one specific symptom (neuropathic pain, cornea verticillata, and/or angiokeratoma), and/or increased globotriaosylsphingosine (Lyso-Gb_3_), and/or an affected family member with a definite diagnosis of Fabry disease. Neuropathic pain was defined as an episode of pain in the hands and/or feet triggered by fever, exercise, or heat, and that had first occurred in childhood or adolescence [7,14]. Angiokeratoma was characterized by clusters of small, dark red spots in characteristic areas, including the bathing trunk area, lips, and umbilicus. Cornea verticillata corresponded to a whorl-like pattern of corneal opacities in the absence of amphiphilic drug use (e.g., amiodarone and chloroquine).

AGAL activity was determined from filter paper dried blood spots, using a validated ultra-high-performance liquid chromatography—tandem mass spectrometry method (Bruker Maxis Impact, Bremen, Germany). Lyso-Gb_3_ was assayed by tandem mass spectrometry on dried blood spots at Centogene AG (Rostock, Germany), Archimed Life Science GmbH (Vienna, Austria), Research Centre for Medical Genetics (Moscow, Russia) or Scientific Center of Children Health (Moscow, Russia). The lyso-Gb_3_ assay cut-off values were ≤1.8 at Centogene AG, ≤3.5 ng/mL at Archimed Life Science GmbH and ≤2.0 ng/mL in Russian laboratories.

For cascade genotyping, the coding exons (1–7) and flanking intronic regions of the *GLA* gene were amplified from purified genomic DNA using PCR. The purified DNA amplicons were sequenced with the Big Dye Terminator version 3.1 Cycle Sequencing Kit (Thermo Fisher Scientific, Waltham, MA, USA). The products were purified with the Big Dye X Terminator Purification Kit (Thermo Fisher Scientific) and resolved using the ABI 3500xL Genetic Analyzer. The data were analyzed using ABI Data Collection software (version 3.0, Thermo Fisher Scientific), Sequencing Analysis software (version 5.2, Thermo Fisher Scientific), and SeqScape software (version 2.6, Thermo Fisher Scientific). Sequences were compared with the reference DNA sequence (GenBank Accession: NM_000169.3).

### Statistical Methods

Continuous variables were quoted as the mean ± standard deviation and/or the median [interquartile range (IQR)], and categorical variables were quoted as the frequency (percentage). Median values were compared using a Wilcoxon test. All statistical analyses were conducted using SPSS Statistics software (version 22, IBM, Armonk, NY, USA).

## 3. Results

### 3.1. Characteristics of the Index Cases

We identified 83 probands (72 males and 11 females) with a definite diagnosis of Fabry disease (Table 1). Thirty-eight (45.8%) patients were undergoing renal replacement therapy for end-stage renal disease and were diagnosed during a nationwide screening program, conducted in Russia’s hemodialysis units. All but three probands were aged 18 or over.

Missense, nonsense, and other pathogenic variants (deletions, splice mutations, or insertions) in the *GLA* gene were found in 50 (60.2%), 15 (18.1%), and 18 (21.7%) patients, respectively. Six variants (c.334C>T, c.644A>G, c.658C>T, c.679C>T, c.680G>A, c.901C>T) were found in more than one family. AGAL activity was absent or very low in 64 (100.0%) males and 4 (40.0%) females. The lyso-Gb_3_ level was above the upper reference limit in all patients and was significantly higher in males than in females.

Most patients presented with early alerting symptoms that were highly suggestive of classic Fabry disease in childhood or adolescence [4,14]. However, the median diagnostic delay was 21 years. At the time of diagnosis, all but one of the patients had advanced renal disease, significant left ventricular hypertrophy, and/or a history of stroke. Following their diagnosis, 39 (46.9%) probands initiated enzyme replacement therapy.

Interestingly, a 42-year-old male patient was found to have a *GLA* pathogenic variant affecting AGAL’s nucleophile residue (c.508G>T, p.D170H). In line with the predicted disruption of the active site, the patient presented with classic Fabry disease and a history of neuropathic pain and hypohidrosis. He progressed to end-stage renal disease and hemodialysis was started at age 39. Fabry disease was only diagnosed during the aforementioned nationwide screening program. His AGAL enzyme activity was low (1.38; normal limit > 1.89); lyso-Gb_3_ levels were not measured. Enzyme replacement therapy was not initiated. The patient died suddenly at the age of 47. No autopsy was performed. The patient had a brother, who was also diagnosed with Fabry disease (no additional information).

Of note, two cases of the p.N215S (p.Asn215Ser) variant were disclosed in this screening performed in end-stage renal disease patients. This could be a mere coincidence since hemizygosity for the *GLA* Ser215 allele is associated with a late-onset form of FD, invariably presenting with cardiac involvement. Cerebrovascular and kidney involvement has also occasionally been reported in some patients [34], but the pathogenic relationship between the incomplete α-galactosidase deficiency and the risk of stroke and of chronic kidney disease is not straightforward [34]. Indeed, the etiologic interpretation of the cerebrovascular and renal complications observed in a few of those patients, and their imputation to FD, is often confounded by the coexistence of major additional risk factors, including long-standing severe and/or inadequately controlled hypertension, type 2 diabetes mellitus, obesity, hyperlipidemia, and tobacco smoking. Next-generation sequencing (NGS) analysis could potentially help identify a second genetic kidney disease beside the late-onset FD associated with the p.N215S variant.

### 3.2. Pedigree Analysis

We investigated 659 family members from one to four generations per family (Table 2). The number of relatives at risk of Fabry disease per proband ranged from 1 to 26 (median: 7). Approximately half of all at-risk family members (331 out of 659) were tested for Fabry disease (AGAL activity in males only, and Lyso-Gb_3_ levels and genetic testing in males and females). The most common reason for lack of testing was poor communication between family members, many of whom were spread out over a large geographic area. Only 30 family members refused genetic testing for personal reasons.

Fabry disease was diagnosed in 165 (49.8%) of the 331 tested family members (51 males and 114 females) (Table 3). Thirty (18.2%) of the 165 newly diagnosed patients were under the age of 18. One hundred and seven patients were symptomatic. The mean ± standard deviation number of affected relatives per proband was 2.0 ± 0.2. Forty-two (25.5%) of the 165 diagnosed family members subsequently initiated enzyme replacement therapy.

## 4. Discussion

In the present study, the construction of family trees and subsequent testing enabled us to diagnose Fabry disease in almost half of the at-risk family members. The mean number of affected relatives was lower in Russia (*n* = 2 per proband) than in a US-based study [35] of 74 patients (*n* = 5 per proband). However, the number of relatives potentially at risk of Fabry disease per proband was relatively low in our study (median: 7), and so our results are not surprising. Family genetic testing was significantly more effective (49.8%) than screening programs in newborns [36] or patients with end-stage renal disease [18,19,20,21,22,23,24,25,26,37,38], unexplained left ventricular hypertrophy [27,28,29,30,39], or stroke [31], which are costly and often have a diagnostic yield below 1% [32]. It is noteworthy that almost 20% of patients newly diagnosed with Fabry disease in this study were under the age of 18, and around 40% of patients were still asymptomatic.

The main barriers to family genetic testing include lack of interest from the physician, the financial cost of testing, and cultural and societal issues. Nevertheless, we were able to test 50.2% of the at-risk family members. The most common obstacle to screening was poor communication between family members, which was frequently related to the family’s geographic spread [33]. Only 4.6% of the family members refused genetic testing for other reasons. It is noteworthy that cost was not an issue in Russia; all tests were provided free of charge both for subjects and medical institutions, while the negative effect of poor infrastructure was minimized by effective logistics and the delivery of dried blood spots from any city to the central laboratory.

Our results suggest that awareness of Fabry disease among physicians is low in Russia. At present, around 250 patients with Fabry disease are registered in Russian reference centers, which would correspond to a prevalence in the general population of approximately 1 per 560,000; the actual prevalence is likely to be ten times or even a hundred times higher [40]. Moreover, almost half of probands were diagnosed with Fabry disease during the nationwide screening of dialysis units. Over 70% of the index patients had a history of early alerting signs and symptoms (i.e., from childhood or adolescence onwards), including neuropathic pain, angiokeratoma, and hypohidrosis/anhidrosis with the occasional occurrence of additional signs such as gastro-intestinal, ENT [41] or respiratory [42] involvement. In most cases, however, a diagnosis of Fabry disease was never considered—not even in patients with overt clinical features and similarly affected relatives. The median time interval between symptom onset and diagnosis was around 20 years for both males and females. It is noteworthy that the data from the Fabry Outcome Survey also suggested that the diagnostic delay for Fabry disease has not significantly fallen over the last decade, despite a trend towards earlier diagnosis in adults and children [43]. Strategies for increasing awareness of Fabry disease among pediatricians are particularly important, given that only three of the probands in this study were under the age of 18 [14].

In the present study, almost 40% of the affected relatives diagnosed with Fabry disease were still asymptomatic. Therefore, genetic testing of all at-risk family members (including males and females) should be encouraged [44]. Ideally, every patient newly diagnosed with Fabry disease should be referred to a medical geneticist for interpretation of the identified genetic variants and a detailed pedigree review [34,45]. However, the pedigree can also be drawn by any healthcare professional who is willing to spend time interviewing index patients and family members, in accordance with international guidelines [34].

## 5. Conclusions

Genetic testing using cascade genotyping in families of index patients identified through symptom presentation or through a screening program can greatly increase the number of patients diagnosed with Fabry disease and can thus facilitate diagnosis and treatment before irreversible organ damage is present.

## Figures and Tables

**Table 1:** Demographic and clinical characteristics of the 83 probands with Fabry disease.

Parameters	Males (*n* = 72)	Females (*n* = 11)	*p*
Age, years	37 [30.5;47.5]	38 [31;64]	0.33
Age at symptom onset, years	10 [6;14]	12 [9;14]	<0.01
Age at diagnosis, years	33 [27;45]	31 [26;41]	0.38
Diagnostic delay, years	21 [13;32]	21 [19;28]	0.05
Early manifestations, *n* (%)			
Neuropathic pain	51 (70.8)	7 (63.6)	0.43
Angiokeratoma	35 (48.6)	3 (27.3)	0.16
Anhidrosis/hypohidrosis	47 (65.3)	3 (27.3)	0.02
Gastrointestinal disorders	19 (26.4)	0	0.05
Renal involvement, *n* (%)			
Albuminuria/proteinuria	62 (86.1)	9 (81.8)	0.49
eGFR 15–60 mL/min/1.73 m^2^	4 (5.6)	2 (18.2)	0.18
End-stage renal disease	38 (52.8)	1 (9.1)	0.01
Dialysis	35 (48.6)	1 (9.1)	0.01
Kidney transplantation	3 (4.2)	0	0.65
Cardiac involvement, *n* (%)			
LVH (echocardiography and/or MRI)	52 (72.2)	3 (27.3)	0.01
Clinically significant arrythmias	8 (11.1)	2 (18.2)	0.39
Cerebrovascular disease, *n* (%)			
White matter lesions on MRI	36/61 (59.0)	3 (27.3)	0.05
Stroke	17 (23.6)	1 (9.1)	0.26
Ophthalmological signs, *n* (%)			
Cornea verticillata	36/51 (70.6)	6 (75.0)	0.25
Cataract	10/51 (19.6)	1 (9.1)	0.37
Pathogenic variants, *n* (%)			
Missense	43 (59.7)	2 (18.2)	0.01
Nonsense	11 (15.2)	4 (36.4)	0.11
Other	18 (25.0)	5 (27.3)	0.15
Low or absent AGAL activity, *n* (%)	64/64 (100.0)	4/10 (40.0)	<0.01
Median lyso-GL3, ng/mL	101	7.2	0.05
Death, *n* (%)	11 (15.3)	0	0.19

Age (in years) is quoted as the median [IQR]; AGAL = α-galactosidase; eGFR = estimated glomerular filtration rate; LVH = left ventricular hypertrophy.

**Table 2:** Pedigree analysis in 83 probands with Fabry disease.

Parameters	Values
Total number of family members	659
Median (range) number of relatives per family	7 (1–26)
Tested for Fabry disease, *n* (%)	331 (50.3)
Not tested for Fabry disease, *n* (%):	328 (49.7)
Poor communication between family members	234
Refused genetic testing	30
Testing is pending	64
Diagnosed with Fabry disease, *n* (%):	165 (49.8)
Males	51
Females	114
Children (age < 18)	30
Symptomatic	107
Mean ± standard deviation number of affected relatives per proband	2.0 ± 0.2

**Table 3:** List of *GLA* pathogenic variants identified in Russian patients diagnosed with Fabry disease (*n* = 97 males and 195 females).

Proband n°	Gender	Age of Diagnosis	A-GAL Activity	Pathogenic *GLA* Variant							Tested Relatives (N)	Diagnosed Relatives (n)
				cDNA Changes	Protein Changes		Type	Consequence	Active Site Residue *	CLINVAR ID		
					One Letter Code	Three Letter Code						
	Female	26	-	c.679C>T	p.R227*	p.Arg227Ter	SNV	Nonsense	-	10733	2	2
2.	Female	35	-	c.679C>T	p.R227*	p.Arg227Ter	SNV	Nonsense	-	10733	3	3
3.	Female	41	-	c.680G>A	p.R227Q	p.Arg227Gln	SNV	Missense	Yes	10732	9	4
4.	Female	31	-	c.946delG	p.V316*	p.Val316Ter	Small deletion	Stop codon	-	NR	1	1
5.	Female	58	-	c.101A>G	p.N34S	p.Asn34Ser	SNV	Missense	-	10724	3	2
6.	Female	28	-	c.658C>T	p.R220*	p.Arg220Ter	SNV	Nonsense	No	167140	14	5
7.	Female	22	-	c.334C>T	p.R112C	p.Arg112Cys	SNV	Missense	No	92550	4	4
8.	Female	26	-	c.901C>T	p.R301*	p.Arg301Ter	SNV	Nonsense	-	92570	7	0
9.	Female	67	-	c.1287_1288dup	p.*430fs	-	Duplication	Frameshift	No	NR	1	0
10.	Female	64	-	c.375delC	p.H125Qfs*5	p.His125Glnfs*5	Small deletion	Frameshift	-	NR	0	0
11.	Female	66	-	c.1000-1G>A	-	-	Consensus Splice Site Mutation	Unknown	-	222111	12	4
12.	Male	49	↓	c.901C>T	p.R301*	p.Arg301Ter	SNV	Nonsense	-	92570	3	0
13.	Male	44	↓↓	c.337delT	p.F113Ffs*17	p.Phel113fs*17	Small deletion	Frameshift	-	NR	0	0
14.	Male	49	↓	c.161T>C	p.L54P	p.Leu54Pro	SNV	Missense	No	NR	2	2
15.	Male	30	↓↓	c.723dupT	p.S241Yfs*8	p.Ser241Tyrfs*8	Small insertion	Frameshift	-	222373	5	3
16.	Male	33	↓	c.658C>T	p.R220*	p.Arg220Ter	SNV	Nonsense	-	167140	7	0
17.	Male	36	↓↓	c.496C>G	p.L166V	p.Leu166Val	SNV	Missense	No	NR	3	0
18.	Male	44	↓↓	c.612G>C	p.W204C	p.Trp204Cys	SNV	Missense	No	NR	0	0
19.	Male	42	↓	c.644A>G	p.N215S	p.Asn215Ser	SNV	Missense	No	10730	5	4
20.	Male	45	↓↓	c.19G>T	p.E7*	p.Glu7Ter	SNV	Nonsense	-	92547	1	1
21.	Male	31	↓	c.1166C>T	p.P389L	p.Pro389Leu	SNV	Missense	No	NR	1	1
22.	Male	49	↓	c.982G>C	p.G328R	p.Gly328Arg	SNV	Missense	No	198053	0	0
23.	Male	52	↓	c.166T>A	p.C56S	p.Cys56Ser	SNV	Missense	No	NR	1	1
24.	Male	23	↓	c.36C>A	p.C12*	p.Cys12Ter	SNV	Nonsense	-	1323004	0	0
25.	Male	33	↓↓	-	p.Y134R	p.Tyr134Arg		Missense	Yes	NR	2	1
26.	Male	42	↓	c.508G>C	p.D170H	p.Asp170His	SNV	Missense	Yes (Nucleophile)	NR	2	1
27.	Male	49	↓↓	c.547G>A	p.G183S	p.Gly183Ser	SNV	Missense	No	222281	1	1
28.	Male	37	↓↓	c.717A>G	p.I239M	p.Ile239Met	SNV	Missense	No	925251	12	3
29.	Male	18	ND	c.128G>T	p.G43V	p.Gly43Val	SNV	Missense	No	928954	9	2
30.	Male	31	ND	c.1056C>T	p.Q386*	p.Gln386Ter	SNV	Nonsense	-	NR	7	7
31.	Male	31	↓↓	c.127G>A	p.G43S	p.Gly43Ser	SNV	Missense	No	290742	3	3
32.	Male	29	↓	c.818T>C	p.F273S	p.Phe273Ser	SNV	Missense	No	222425	6	0
33.	Male	30	↓↓	c.370_377del	p.V124Qfs*14	p.Val124Glnfs*14	Small deletion	Frameshift	-	NR	4	1
34.	Male	26	↓↓	c.548-2A>G	-	-	Consensus Splice site Mutation	Unknown	-	92554	11	5
35.	Male	16	ND	c.1085_1098del14	p.P362Hfs*8	p.Pro362Hisfs*8	Small deletion	Frameshift	-	NR	7	1
36.	Male	38	↓↓	c.422C>T	p.T141I	p.Thr141Ile	SNV	Missense	No	285570	2	2
37.	Male	29	↓↓	c.550T>G	p.Y184D	p.Tyr184Asp	SNV	Missense	No	997944	6	6
38.	Male	14	↓↓	c.539_547del9insC	p.L180_G183del9insC	p.Leu180_Glydel9insC	Small deletion/small insertion	Frameshift	-	NR	6	2
39.	Male	48	↓↓	c.145C>G	p.R49G	p.Arg49Gly	SNV	Missense	-	NR	1	1
40.	Male	18	↓↓	c.513A>G	p.K168R	p.Lys168Arg	SNV	Missense	Yes	NR	4	1
41.	Male	12	↓↓	c.1163T>A	p.L388H	p.Leu388His	SNV	Missense	No	NR	4	1
42.	Male	30	↓↓	c.902G>T	p.R301L	p.Arg301Leu	SNV	Missense	No	NR	7	5
43.	Male	48	↓↓	c.493G>T	p.D165Y	p.Asp165Tyr	SNV	Missense	No	NR	3	0
44.	Male	52	↓↓	c.203T>C	p.L68P	p.Leu68Pro	SNV	Missense	No	NR	7	0
45.	Male	53	↓↓	c.334C>T	p.R112C	p.Arg112Cys	SNV	Missense	No	92550	4	4
46.	Male	7	ND	c.782G>T	p.G261V	p.Gly261Val	SNV	Missense	No	222387	3	3
47.	Male	27	↓↓	c.161T>C	p.L54P	p.Leu54Pro	SNV	Missense	No	NR	11	0
48.	Male	15	↓↓	c.334C>T	p.R112C	p.Arg112Cys	SNV	Missense	No	92550	2	0
49.	Male	21	↓↓	c.844A>C	p.T282P	p.Thr282Pro	SNV	Missense	No	NR	4	2
50.	Male	54	↓↓	c.109G>A	p.A37T	p.Ala37Thr	SNV	Missense	No	1324470	2	0
51.	Male	31	↓↓	c.847C>T	p.Q283*	p.Gln283Ter	SNV	Nonsense	-	180843	9	3
52.	Male	30	↓↓	c.1277_1278delAA	p.K426Rfs*	p.Lys426Argfs*	Small deletion	Frameshift	-	10772	3	2
53.	Male	42	↓	c.679C>T	p.R227*	p.Arg227Ter	SNV	Nonsense	-	10733	3	0
54.	Male	42	↓↓	c.1197G>A	p.W399*	p.Trp399Ter	SNV	Nonsense	-	NR	2	1
55.	Male	48	↓↓	c.1021G>A	p.E341K	p.Glu341Lys	SNV	Missense	No	222125	14	9
56.	Male	30	ND	c.444T>G	p.S148R	p.Ser148Arg	SNV	Missense	No	633251	7	5
57.	Male	?	↓↓	c.572T>A	p.L191Q	p.Leu191Gln	SNV	Missense	No	NR	0?	0?
58.	Male	52	↓↓	c.1072_1074delGAG	p.E358del	p.Glu358del	Small in frame deletion	Unknown	-	180844	1	0
59.	Male	31	↓↓	c.442_450delAGTTTTGGA	p.S148_G150del	p.Ser148_Gly150del	Small in frame deletion	Unknown	-	NR	8	5
60.	Male	19	↓↓	c.804A>C	p.L268F	p.Leu268Phe	SNV	Missense	No	NR	1	1
61.	Male	51	↓	c.717A>G	p.I239M	p.Ile239Met	SNV	Missense	No	925251	2	1
62.	Male	50	↓	c.758T>C	p.I253T	p.Ile253Thr	SNV	Missense	No	180021	2	1
63.	Male	36	↓↓	c.1133G>A	p.C378Y	p.Cys378Tyr	SNV	Missense	No	NR	17	12
64.	Male	31	↓	c.786delG	p.W262*	p.Trp262Ter	Small deletion	Stop codon	-	NR	3	3
65.	Male	15	↓↓	c.521G>A	p.C174Y	p.Cys174Tyr	SNV	Missense	No	NR	4	3
66.	Male	32	ND	c.227T>C	p.M76T	p.Met76Thr	SNV	Missense	No	NR	5	4
67.	Male	41	↓	c.1025G>A	p.R342Q	p.Arg342Gln	SNV	Missense	No	10742	5	1
68.	Male	54	↓↓	c.658C>T	p.R220*	p.Arg220Ter	SNV	Nonsense	-	167140	2	2
69.	Male	20	↓	c.949del	p.A350Vfs*2	p.Ala350Valfs*2	Small deletion	Frameshift	-	NR	3	3
70.	Male	20	↓↓	c.658C>T	p.R220*	p.Arg220Ter	SNV	Nonsense	-	167140	3	0
71.	Male	43	↓↓	c.901C>T	p.R301*	p.Arg301Ter	SNV	Nonsense	-	92570	1	1
72.	Male	38	↓↓	c.1049delC	p.A350Vfs*2	p.Ala350Valfs*2	Small deletion	Frameshift	-	92538	2	2
73.	Male	24	↓↓	c.1033_1034del	p.S345Rfs*29	p.Ser345Argfs*29	Small deletion	Frameshift	-	92538	0	0
74.	Male	48	↓↓	c.983G>C	p.G328A	p.Gly328Ala	SNV	Missense	-	10740	1	1
75.	Male	46	↓↓	c.644A>G	p.N215S	p.Asn215Ser	SNV	Missense	No	10730	1	1
76.	Male	39	↓↓	c.869T>C	p.M290T	p.Met290Thr	SNV	Missense	No	684855	1	3
77.	Male	30	↓↓	c.551A>G	p.Y184C	p.Tyr184Cys	SNV	Missense	No	NR	2	2
78.	Male	28	↓↓	c.614C>G	p.P205R	p.Pro205Arg	SNV	Missense	No	NR	1	1
79.	Male	17	ND	c.44C>A	p.A15E	p.Ala15Glu	SNV	Missense	No	NR	1	1
80.	Male	57	↓↓	c.269G>A	p.C90Y	p.Cys90Tyr	SNV	Missense	No	NR	2	2
81.	Male	15	ND	c.680G>A	p.R227Q	p.Arg227Gln	SNV	Missense	Yes	10732	6	4
82.	Male	34	↓↓	c.671A>G	p.N224S	p.Asn224Ser	SNV	Missense	No	222365	3	2
83.	Male	25	↓	c.269G>A	p.C90Y	p.Cys90Tyr	SNV	Missense	No	NR	2	0

* For Missense variant only. ND: not done, ↓: significantly below cut-off level, ↓↓: zero or close to zero. NR = not reported.

## Data Availability

Data supporting reported results can be provided on request.
